# Identification of a disease-associated germline mutation (A64T) of the ring finger protein 186 gene (*RNF186*) in Korean patients with ulcerative colitis

**DOI:** 10.1016/j.gendis.2024.101438

**Published:** 2024-10-06

**Authors:** Sang-Hun Park, Sae-Young Won, Yong-Chan Kim

**Affiliations:** Department of Biological Sciences, Andong National University, Andong 36729, Republic of Korea

Ulcerative colitis is a major type of inflammatory bowel disease characterized by chronic idiopathic mucosal inflammation in the rectum to the colon. Patients with ulcerative colitis exhibit a life expectancy of five years shorter than the general population, and within five years of diagnosis, 7% undergo colectomy. As of 2023, the global prevalence of ulcerative colitis is estimated to be higher than five million cases, and the incidence rate is increasing. However, the precise etiology remains unclear.^1^ A previous genome-wide association study (GWAS) reported that the ring finger protein 186 gene (*RNF186*) is a potent pathogenesis-related factor of ulcerative colitis. RNF186 protein plays a pivotal role in intestine homeostasis through the regulation of the expression of occludin, a major gut barrier component, by polyubiquitination. Moreover, the colitis symptoms of *RNF186* gene knockout mice were more severe compared with wild-type mice. In addition, the two genetic variants A64T (rs41264113) and R179X (rs36095412) that result in an altered form of RNF186 protein were shown to be associated with the pathomechanism of ulcerative colitis in a Caucasian population.^2^^,^^3^ Additionally, both the A64T and R179X variants result in changes in amino acids in the protein. However, the A64T variant confers susceptibility to ulcerative colitis, while the R179X variant confers resistance to ulcerative colitis.

These conflicting results suggest the need for further exploration of the effects of these variants in colitis patients. To evaluate the associations between the two genetic variants, A64T (rs41264113) and R179X (rs36095412), and ulcerative colitis in clinical patients, we investigated the genotype and allele distributions of these variants in the *RNF186* gene in Korean ulcerative colitis patients and matched controls using amplicon sequencing. In addition, we performed an association analysis to determine the relationship between the genetic variants and ulcerative colitis. Furthermore, we collected genetic information on A64T (rs41264113) and R179X (rs36095412) in East and South Asian populations, which are similar ethnic backgrounds, from 1000 Genomes Project to evaluate the associations between the genetic variants and susceptibility to ulcerative colitis.

A total of 156 individuals were included in the association analysis (Table S1). The 79 ulcerative colitis patients included 28 females and 51 males. The 77 healthy controls included 31 females and 46 males. The two groups exhibited a similar distribution of sex (*p* = 0.535). The mean age at diagnosis of ulcerative colitis patients was 40.00 ± 15.27 years, and the mean age of healthy controls at sample collection was 38.69 ± 13.58 years. The mean age was similar in the two groups (*p* = 0.571).

To evaluate the associations between the two genetic variants A64T (rs41264113) and R179X (rs36095412) and ulcerative colitis, we performed amplicon sequencing and carried out a case–control association study. The detailed location of the two genetic variants is shown in Figure 1A. Noticeably, the A64T variant was observed only in two ulcerative colitis patients (Fig. 1B), with low genotype (2.5%) and allele (1.3%) frequencies (Table S2). These two ulcerative colitis patients, females aged 25 and 45, respectively, had no comorbidities (Fig. 1C). We did not find R179X variants in ulcerative colitis patients and matched controls in the Korean population (Table S2). In addition, we did not observe R179X variants in the South and East Asian population, which has a similar ethnic background to the Korean population.

**Figure 1:**
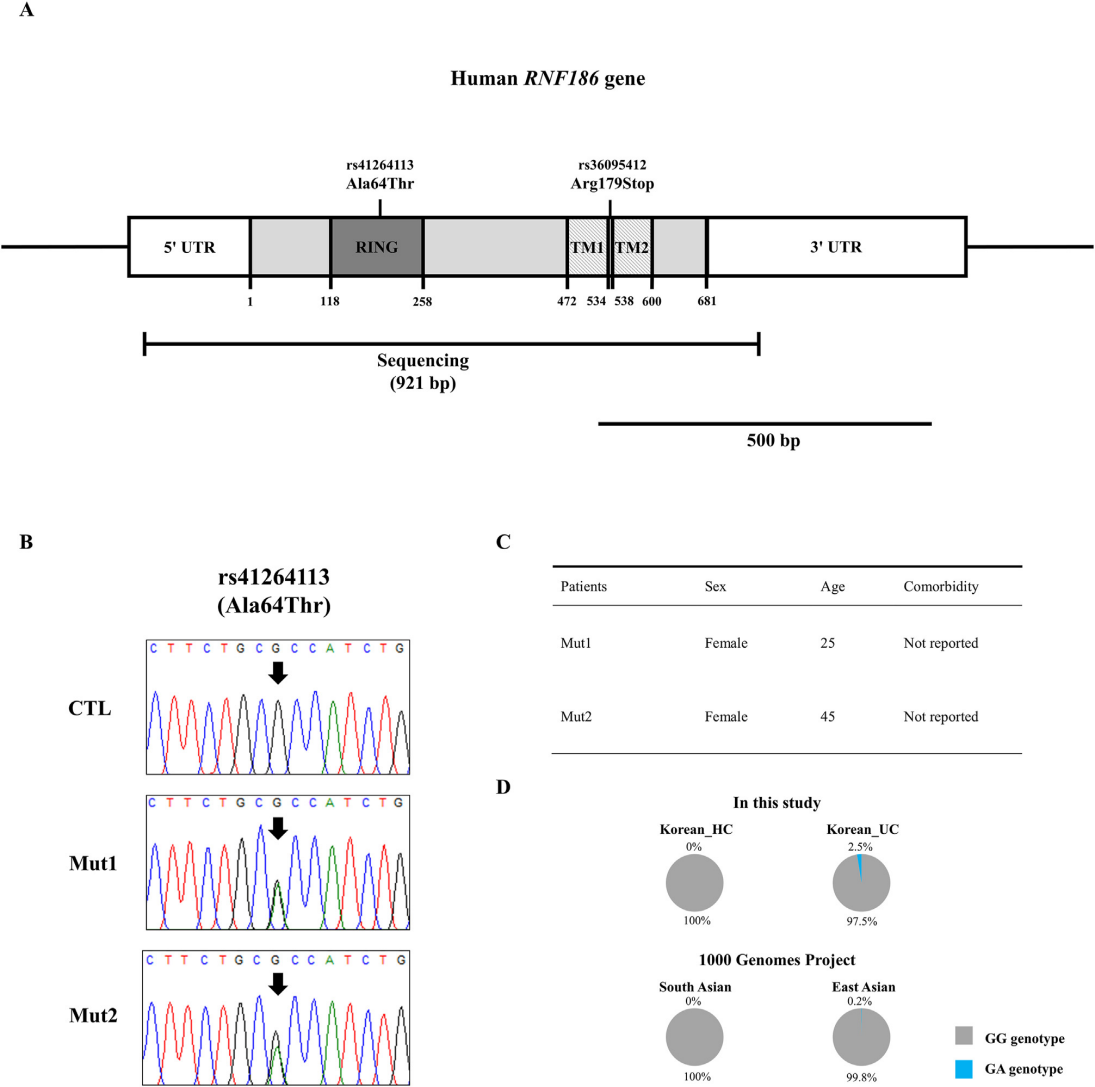
Identification of A64T germline mutation in *RNF186* gene in ulcerative colitis patients. **(A)** Schematic map of *RNF186* gene and the locations of the two genetic variants, A64T (rs41264113) and R179X (rs36095412). The open reading frame (ORF) is indicated by a gray block (681 bp). The 5′ untranslated region (UTR) and 3′ UTR are indicated by white blocks. The edged horizontal bar indicates the regions sequenced (921 bp). **(B)** Electropherograms of the A64T germline mutation in *RNF186* gene in ulcerative colitis patients. **(C)** Detailed information on two ulcerative colitis patients carrying the A64T germline mutation. **(D)** Comparison of the genotype distribution of the A64T germline mutation of the *RNF186* gene in Korean and South/East Asian populations. *RNF186*, ring finger protein 186 gene; RING, ring finger domain; TM1, transmembrane domain 1; TM2, transmembrane domain 2; CTL, healthy controls; Mut1, ulcerative colitis patient 1 carrying A64T germline mutation; Mut2, ulcerative colitis patient 2 carrying A64T germline mutation; Korean_HC, Korean healthy controls; Korean_UC, Korean ulcerative colitis patients.

To evaluate the association of A64T (rs41264113) genetic variants with susceptibility to ulcerative colitis in larger populations, we collected data on the genotype and allele distributions of the A64T (rs41264113) variant in South and East Asian populations, which have a similar ethnic background to the Korean population from the 1000 Genomes Project database. A64T heterozygotes are highly observed among Korean ulcerative colitis patients (2.5%) compared with South (0.2%) and East (0%) Asian populations (Fig. 1D and Table S2). Compared with ulcerative colitis patients, South Asian individuals showed significantly different genotype and allele distributions of the A64T (rs41264113) variant of the *RNF186* gene (*p* < 0.05). In addition, East Asian patients showed significantly different allele distributions of A64T (rs41264113) in *RNF186* gene compared with ulcerative colitis patients (*p* < 0.05) (Table S2).

In the present study, we investigated the genetic distributions of the two genetic variants A64T (rs41264113) and R179X (rs36095412) in the *RNF186* gene and performed association analysis to identify the relationships between these genetic variants and susceptibility to ulcerative colitis. The R179X variant was not found in either ulcerative colitis patients or matched controls. The A64T genetic variant was found only in ulcerative colitis patients (Table S2). In addition, the A64T variant showed significantly different genetic distributions between Korean ulcerative colitis patients and East/South Asian populations. These results indicate that the A64T genetic variant may be a genetic susceptibility factor for ulcerative colitis in the Asian population. Furthermore, because of the very low frequency of the A64T variant in control populations (<1 %), the A64T variant is better classified as a mutation rather than a polymorphism in the Asian population. To date, the germline mutations that cause ulcerative colitis have not been identified. In the present study, we report the *RNF186* germline mutation as an ulcerative colitis–related germline mutation. Further study of this variant in a larger group is highly desirable to validate the causality of this mutation. Both variants result in changes in the structure and function of the protein; however, the effects of each variant are controversial.^1^^,^^2^ These conflicting results suggest the need for further validation of the effects of these variants on clinical outcomes in ulcerative colitis patients.

The GWAS analysis follows the “common disease–common variant” hypothesis.^5^ Thus, rare variants with a minor allele frequency of less than 5% are removed during the quality control process to conduct further analysis. Previous studies on the A64T and R179X variants were performed based on GWAS.^4^ However, the minor allele frequencies of the A64T and R179X variants were less than 5% in all ethnic groups (1000 Genomes Project). This suggests that there may be other genetic variants associated with susceptibility to ulcerative colitis in addition to the A64T and R179X variants. Therefore, further fine mapping of the *RNF186* gene is highly desirable. In addition, previous GWAS found that the rs3806308, rs1317209, and rs6426833 variants are associated with colitis.^4^ These genetic variants are located in regions adjacent to the *RNF186* gene, OTU deubiquitinase 3 (*OTUD3*), and phospholipase A2 group IIE (*PLA2G2E*) genes. Thus, further analysis of the relationship between the *OTUD3* and *PLA2G2E* genes and ulcerative colitis is highly recommended.

In conclusion, we found the A64T variant of the *RNF186* gene only in Korean ulcerative colitis patients. Additionally, we identified significantly different distributions of the A64T variant of the *RNF186* gene between Korean ulcerative colitis patients and South/East Asian populations. In contrast, we did not observe the R179X variant of the *RNF186* gene in either ulcerative colitis patients or matched healthy controls. To the best of our knowledge, this is the first report of a disease-related germline mutation of the *RNF186* gene in Korean patients with ulcerative colitis.

## CRediT authorship contribution statement

**Sang-Hun Park:** Writing – original draft, Methodology, Investigation, Formal analysis, Data curation. **Sae-Young Won:** Writing – original draft, Validation, Methodology, Investigation, Funding acquisition. **Yong-Chan Kim:** Writing – review & editing, Writing – original draft, Validation, Supervision, Funding acquisition.

## Ethics declaration

All experimental procedures were approved by the Institutional Review Board of Andong National University and in accordance with the 1964 Helsinki Declaration and its later amendments (IRB No. 1040191-202306-BR-002-01). All information on samples and related data was anonymized prior to the analysis. All materials derived were obtained (with informed consent) under Institutional Review Board-approved protocols.

## Funding

This work was supported by the National Research Foundation of Korea grant funded by the Korean government (The South Korean Ministry of Science and ICT) (No. 2022R1C1C2004792). This research was supported by the Basic Science Research Program through the National Research Foundation of Korea funded by the Ministry of Education (No. RS-2023-00273199). This work was also supported by a research grant from Andong National University.

## Conflict of interests

The authors declare no conflict of interest.

## Data Availability

All data are available from the corresponding authors upon reasonable request.
