# Supplementary material for: Identification of a disease-associated germline mutation (A64T) of the ring finger protein 186 gene (RNF186) in Korean patients with ulcerative colitis
Source: Genes Dis. 2024 Oct 6;12(4):101438. doi: 10.1016/j.gendis.2024.101438 (PMC11995066; doi:10.1016/j.gendis.2024.101438)
Supplement: Multimedia component 1 [file mmc1.docx]

**Supplementary Data**

**Supplementary materials and methods**

**Blood samples**

Blood samples of ulcerative colitis patients were provided by the Chungbuk and Kyungpook National University hospital biobank, a member of the Korea Biobank Network. The blood samples of age- and sex-matched healthy controls were provided from the Ajou University Hospital Biobank, a member of the Korea Biobank Network.

**Ethical statements**

All experimental procedures were approved by the Institutional Review Board (IRB) of Andong National University and in accordance with the 1964 Helsinki Declaration and its later amendments (IRB No. 1040191-202306-BR-002-01). All information on samples and related data were anonymized prior to the analysis. The Biospecimens and data used in this study were provided by the Biobank of Chung Buk University Hospital, a member of Korea Biobank Network. The biospecimens and data used for this study were provided by the Biobank of Ajou University Hospital, a member of Korea Biobank Network. The biospecimens and data used for this study were provided by the Biobank of Korea-Kyungpook National University Hospital (KNUH), a member of the Korea Biobank Network. All materials derived from the National Biobank of Korea-KNUH were obtained (with informed consent) under IRB-approved protocols (project No.2024-ER0506-00).

**Genomic DNA extraction**

The genomic DNA was isolated from 100 µl buffy coat of 77 controls and 79 ulcerative colitis patients using a Genomic DNA prep Kit (BIOFACT, Daejeon, Republic of Korea) following the manufacturer’s instructions. The isolated genomic DNA was measured for concentration and purity by 1% agarose gel electrophoresis and NanoDrop analysis.

**Genetic analysis**

Polymerase chain reaction (PCR) was conducted using the BioFACT™ Taq DNA Polymerase (BIOFACT, Daejeon, Republic of Korea) following the manufacturer’s protocols with minor modification (annealing at 56°C, 30 s). The PCR reaction contained gene-specific forward (5’-TCTGGGAGAGAGGAGTGTCAG-3’) and reverse (5’-ACCCCTTACCCTGTTCATTGT-3’) primers for the human *RNF186* gene (Gene ID: 54546) designed by Primer3Plus program (<https://www.bioinformatics.nl/cgi-bin/primer3plus/primer3plus.cgi>). The PCR amplicons were analyzed by gel electrophoresis and obtained using a FavorPrep GEL/PCR purification mini kit (FAVORGEN, Ping Tung, Taiwan, China). Sequencing was performed using an ABI 3730 sequencer (ABI, Foster City, CA, USA), and sequence analysis was conducted using Finch TV software (Geospiza Inc., Seattle, WA, USA).

**Genetic information on South and East Asian populations**

The genotype and allele frequencies of A64T (rs41264113) and R179X (rs36095412) in *RNF186* in the South and East Asian populations were collected from the 1000 Genomes Project (accessed on 2024 May 22)

**Statistical analysis**

All statistical analyses were performed using SAS version 9.4 (SAS Institute Inc., USA). The differences in genotype and allele distributions of the *RNF186* gene between ulcerative colitis patients and matched control populations were compared using Fisher's exact test. The sex distribution and mean age between the two groups were compared by χ2 and Student’s t-test, respectively.

**Supplementary Table 1** The detailed information of the study population.

| Characteristics |  | Ulcerative colitis patients | Controls | *P*-value |
| --- | --- | --- | --- | --- |
| Number |  | 79 | 77 | - |
| Age |  | 40.00±15.27 | 38.69±13.58 | 0.571 |
| Sex (*n*, %) | Male | 51 (64.56) | 46 (59.74) | 0.535 |
|  | Female | 28 (35.44) | 31 (40.26) | - |

**Supplementary Table 2** Comparison of genotype and allele distributions between healthy controls and ulcerative colitis patients.

| Variants |  | Genotype frequency, *n* (%) | | | *P*-value | Allele frequency, *n* (%) | | *P*-value |
| --- | --- | --- | --- | --- | --- | --- | --- | --- |
|  |  | GG | GA | AA |  | G | A |  |
| c.190G>A (Ala64Thr) | East Asian | 503 (99.8) | 1 (0.2) | 0 (0) | 0.5 | 1007 (99.9) | 1 (0.1) | **<0.05** |
| rs41264113 | South Asian | 489 (100) | 0 (0) | 0 (0) | **<0.05** | 978 (100) | 0 (0) | **<0.05** |
|  | Controls_Koreans | 77 (100) | 0 (0) | 0 (0) | 0.497 | 154 (100) | 0 (0) | 0.498 |
|  | Ulcerative colitis patients_Koreans | 77 (97.5) | 2 (2.5) | 0 (0) | - | 156 (98.7) | 2 (1.3) | - |
|  |  |  |  |  |  |  |  |  |
|  |  | CC | CT | TT |  | C | T |  |
| c.535C>T (Arg179Stop) | East Asian | 2597 (0) | 0 (0) | 0 (0) | 1.0 | 5194 (0) | 0 (0) | 1.0 |
| rs36095412 | South Asian | 2412 (0) | 0 (0) | 0 (0) | 1.0 | 4824 (100) | 0 (0) | 1.0 |
|  | Controls_Koreans | 77 (100) | 0 (0) | 0 (0) | 1.0 | 154 (100) | 0 (0) | 1.0 |
|  | Ulcerative colitis patients_Koreans | 79 (100) | 0 (0) | 0 (0) | - | 158 (100) | 0 (0) | - |
